# Blind data hiding technique using the Fresnelet transform

**DOI:** 10.1186/s40064-015-1534-1

**Published:** 2015-12-30

**Authors:** Nazeer Muhammad, Nargis Bibi, Zahid Mahmood, Dai-Gyoung Kim

**Affiliations:** 1 Department of Applied Mathematics, Hanyang University, Ansan, 426-791 South Korea; 2 Department of Mathematics, COMSATS Institute of Information Technology, Wah Cantt, Pakistan; 3 School of Computer Science, University of Manchester, Manchester, UK; 4 Department of Computer Science, Fatima Jinnah Women University, Rawalpindi, Pakistan; 5 Department of Electrical and Computer Engineering, North Dakota State University, Fargo, USA

**Keywords:** Blind data hiding, Fresnelet transform, Image encryption, Wavelet transform

## Abstract

A new blind data hiding scheme is proposed in which data is decomposed using the Fresnelet transform. The inverse Fresnelet transform is performed on decomposed subbands by choosing different key parameters, and the coded pattern of the information data is obtained. This coded pattern is embedded into particular subbands of the cover image using the wavelets. The proposed method has good imperceptibility and large capacity of the information embedded data. Using the Fresnelet transform with a family of wavelet transforms makes the scheme more efficient in terms of extracted accuracy of hidden information. Moreover, the hidden data can be recovered without access to the original cover data. The proposed method is used to resolve privacy and security issues raised with respect to emerging internet applications for the effective handling of confidential data.

## Background

Data hiding is an integral part of Internet technologies. Internet users frequently need to store, send, or receive private information. As the amount of information placed on the internet increases, so does the need for protection from unwanted reconnaissance, embezzlement, and fictitious propaganda. In this context, images are subject to changes which are unidentifiable by the human visual system. The most common way to avoid any discrepancy is to transform the data into an encrypted form. The resulting data can be understood only by those who know how to return it to its original form with special key parameters. Data encryption enables data confidentiality, integrity, and data authentication. For secure digital communication, it is important to conceal confidential data into some digital cover media such that it does not reveal its original contents (Petitcolas et al. 1999). This protection can be achieved using a digital data hiding technique which encrypts the meaningful information into dummy data and then embed it into other data (called the host or cover data). This scheme has been developed for information security and is strongly based on cryptography and steganography (Zaidan et al. 2010).

Cryptography and steganography are both used for data confidentiality. Cryptographic techniques can be applied to an information hiding scheme in order to encrypt the secret data prior to embedding it. It keeps the contents of a message secret by scrambling the original information into uncorrelated data, whereas steganography conceals the original information within some cover data (Dickman 2007). Combining these two schemes into one hybrid system is likely to provide even better security and confidentiality (Wu et al. 2005).

One common way of data hiding leads to manipulation of the Least Significant Bit (LSB) plane. Some examples of LSB schemes have been presented in Yang et al. (2009), achieving both high capacity and low perceptibility. However, in this embedding scheme a kind of structural asymmetry (while hiding the data, the even pixels remain constant and odd pixels increase) is introduced such that LSB makes the disclosure of hidden message very easy (Ker 2007).

Ashourian and Ho (2005) proposed a methodology for hiding a low resolution grayscale information image into another grayscale cover image with higher resolution. They adopted blind detection by embedding a secret grayscale image. However, the algorithm is limited to communication of high resolution secret images that are one-fourth the size of the original cover image. Chang et al. extended Iwata et al.’s idea (Chang et al. 2007) and presented a lossless stenographic scheme for hiding significant information in each block of quantized DCT coefficients in JPEG images (Lin and Shiu 2009). However, this scheme can only embed secret bits with very having very small capacity (Lin and Shiu 2009). Lin and Shiu (Conway 2003) designed a two-layer data hiding scheme using Chang et al.’s (2007) method for DCT-based images. This scheme performs better than Chang scheme (Chang et al. 2007) in terms of hiding, but the size of the hidden data is still less than 70 k bits on average.

Based on the techniques described above, it has been concluded that two main factors affecting information hiding are the visual quality of stegano-images and embedding capacity (or payload) (Chih-Yang et al. 2008; Dhavale et al. 2014). An information hiding scheme with low image distortion is more secure than that with high distortion because it does not raise any suspicions of adversaries. The second factor, embedding capacity with high payload is preferred because more confidential data can be efficiently transferred for a wide range of applications such as defense organizations, military, intelligence agencies, and medical imaging (Anand and Niranjan 1998).

Data hiding techniques can be categorized as blind or non-blind techniques. In a blind data hiding technique, the hidden information can be extracted from the embedded media without access to the original cover media, making it more secure. In a non-blind data hiding technique, the original cover media has to be available at the extraction stage. Blind extraction has broader applicability because assuming the original cover media will be available is not always realistic. However, it is more difficult to implement the blind data hiding technique, especially when the cover media is considered as an image since the cover image and the embedded information cannot be easily decoupled from the processed data at the receiver end (Pan et al. 2009).

To our knowledge, no prior work has discussed the application of the Fresnelet transform in conjunction with blind data hiding steganography. We previously proposed a non-blind data hiding technique (Nazeer et al. 2013). In this paper, we use the blind data hiding technique by blending both steganography and cryptography for handling the large amount of the secret data.

The combination of the forward and backward Fresnelet has been used in order to build dummy sets of the secret data rather than using only the wavelet or the Fresnel transform (Liebling and Unser 2004; Maheswari and Hemanth 2015). At first place, the Fresnelet transform is designed for (Xuan et al. 2002) reconstructing the digital holography with high resolution of pixel values of the target objects (Nazeer et al. 2013). Therefore, it can be useful to decompose and reconstruct the digital multimedia content for data hiding purpose to provide more data security and reliability (Zhou et al. 2014). Moreover, by the Fresnelet transform the high resolution information can be extracted from an embedded data in ongoing digital communications (Liebling et al. 2003). The key factors of the Fresnelet transform are used to analyze the well-defined understanding of the contents of concealed communication. Preserving the high resolution of the secret image data at the embedding stage and retrieving the accurate resolution at the extraction stage are keys to a secure communication of the concealing contents. A multi-scale distribution of the secret information using the robust key parameters is obtained using the Fresnelet transform domain yielding high security and better privacy. One of the main features of the proposed method is inaccessibility to get the concealed data without the exact keys, even if an attacker is aware of the hiding algorithm.

Later part of this paper is ordered as follows. “Fresnelet transform encryption” provides a theoretical explanation of the Fresnelet transform. The data encryption process is described in the same section. “Proposed method” describes the proposed method of using Fresnelet transforms in a blind data hiding algorithm. “Simulation and evaluation” presents the simulations and results, and “Conclusions” concludes the paper.

## Fresnelet transform encryption

The complex wave propagation is generated using the Fresnel transform diffraction through the propagation phenomena (Zhou et al. 2014; Nazeer and Kim 2013). When the Fresnel transform is applied to a multi-resolution bases of the wavelet, it produces the Fresnelet transform basis. These basis have been used to reconstruct the digital hologram with varying sets of parameters. These parameters are composed on value of the resolution scale, the wavelength, and the distance between the propagating objects and the observing plane. Fresnelet transform has been presented to simulate the approximation model of the monochromatic waves propagation. In this regard, one-dimensional data propagation is shown with the Fresnel transform model for a function $f \in L_{2} \left( {\mathbb{R}} \right)$ that can be represented as the convolution integral:

1 $$ \tilde{f}_{\tau } \left( x \right) = \left( {f \times k_{\tau } } \right)\left( x \right)\,{\text{with }}\,k_{\tau } \left( x \right) = \frac{1}{\tau }exp\left( {i\pi \frac{{x^{2} }}{{\tau^{2} }}} \right) $$

where $k_{\tau } \left( x \right)$ is the one-dimensional kernel. And the normalizing parameter $\tau > 0$ depending on the distance $d$ and on the wavelength $\lambda$ as follow:

$$ \tau = \sqrt {\lambda d} $$

In addition, two-dimensional data propagation is shown by using the tensor product of the $k_{\tau } \left( x \right)$, for $f \in L_{2} \left( {{\mathbb{R}}^{2} } \right)$,

$$ \tilde{f}_{\tau } \left( {x,y} \right) = \left( {f \times K_{\tau } } \right)\left( {x,y} \right)\,{\text{with }}\,K_{\tau } \left( {x,y} \right) = k_{\tau } \left( x \right)k_{\tau } \left( y \right). $$

where $K_{\tau } \left( {x,y} \right)$ is the separable kernel used to extend the Fresnel transform’s one- dimensional case readily to two-dimensional case (Zhou et al. 2014). Among the various useful properties of the Fresnel transform, the unitary property is the prominent one, so that the given data can be facilitated to obtain the perfect reconstruction.

The separable extension of the one-dimensional wavelet into two-dimensional wavelet can also be obtained. The Riesz basis for $L_{2} \left( {\mathbb{R}} \right)$, can be defined in terms of a two parameter family $\left\{ {\psi_{j,l} } \right\}_{{j, l \in {\mathbb{Z}}}}$ on $L_{2} \left( {\mathbb{R}} \right)$ using the wavelet transform as convolution integrals, where

2 $$ \left\{ {\psi_{j,l} \left( x \right) = 2^{j/2} \psi \left( {2^{j} x - l} \right)} \right\}_{{j, l \in {\mathbb{Z}}}} . $$

An orthonormal basis for the Haar wavelet can also be generated for $L_{2} \left( {\mathbb{R}} \right)$. It is also known as the simplest form of a wavelet that can attain multiresolution decomposition and the perfect reconstruction of the given data as well (Kang and Aoki 2005). Furthermore, the Fresnelet basis is obtained using the Fresnel transform to the Haar wavelet as follows:

3 $$ \left\{ {\left( {\psi_{j,l} } \right)_{{\tilde{\tau }}} } \right\}_{{j, l \in {\mathbb{Z}}}} \,\,{\text{with}}\,\,\left( {\psi_{j,l} } \right)_{{\tilde{\tau }}} \left( x \right) = 2^{j/2} \tilde{\psi }_{{2^{j} \tau }} \left( {2^{j} x - l} \right). $$

An orthonormal Fresnelet basis can be attained for fixed $\tau$, by letting $\varTheta_{j,l} \left( x \right) = \left( {\psi_{j,l} } \right)_{{\tilde{\tau }}} \left( x \right)$, as follows:

4 $$ f = \mathop \sum \limits_{j,l}  c_{j,l}  \varTheta_{j,l} \,{\text{with}}\,c_{j,l} = \left\langle {f,\varTheta_{j,l} } \right\rangle . $$

The Fresnelet coefficients are represented by $c_{j,l}$ in (4). Since the separable nature can be used to extend the Fresnelet transform’s one-dimensional domain readily to two-dimensional domain. Following this, we may obtain four possible combinations of the tensor product $\gamma_{\tau }^{{\left( {ll} \right)}} ,\, \gamma_{\tau }^{{\left( {lh} \right)}} ,\, \gamma_{\tau }^{{\left( {hl} \right)}} , \,{\text{and}} \,\gamma_{\tau }^{{\left( {hh} \right)}} ,$ or generating the lower–lower subband, lower-high detail, high-lower detail, and high–high detail subbands, respectively, as follow:

5 $$ \gamma_{\tau }^{{\left( {ll} \right)}} = \left( {\phi_{j,l} } \right)_{{\tilde{\tau }}} \left( x \right)\left( {\phi_{j,l} } \right)_{{\tilde{\tau }}} \left( y \right), $$

6 $$ \gamma_{\tau }^{{\left( {lh} \right)}} = \left( {\phi_{j,l} } \right)_{{\tilde{\tau }}} \left( x \right)\left( {\psi_{j,l} } \right)_{{\tilde{\tau }}} \left( y \right), $$

7 $$ \gamma_{\tau }^{{\left( {hl} \right)}} = \left( {\psi_{j,l} } \right)_{{\tilde{\tau }}} \left( x \right)\left( {\phi_{j,l} } \right)_{{\tilde{\tau }}} \left( y \right), $$

8 $$ \gamma_{\tau }^{{\left( {hh} \right)}} = \left( {\psi_{j,l} } \right)_{{\tilde{\tau }}} \left( x \right)\left( {\psi_{j,l} } \right)_{{\tilde{\tau }}} \left( y \right), $$

where the $\phi$ is representing the scaling function and $\psi$ is representing the wavelet functions, respectively. Following these functions, the (5) is establishing a low-pass filter and the (6), (7), (8) are establishing high-pass filters. By using the above basis functions to data *f*, the four Fresnelet coefficients are as follows:

$$ f_{\tau ,d}^{{\left( {ll} \right)}} = \left\langle {f,\gamma_{\tau }^{{\left( {ll} \right)}} } \right\rangle ,\,\,f_{\tau ,d}^{{\left( {lh} \right)}} = \left\langle {f, \gamma_{\tau }^{{\left( {lh} \right)}} } \right\rangle , $$

$$ f_{\tau ,d}^{{\left( {hl} \right)}} = \left\langle {f,\gamma_{\tau }^{{\left( {hl} \right)}} } \right\rangle ,\,\,f_{\tau ,d}^{{\left( {hh} \right)}} = \left\langle {f,\gamma_{\tau }^{{\left( {hh} \right)}} } \right\rangle , $$

**Fig. 1:**
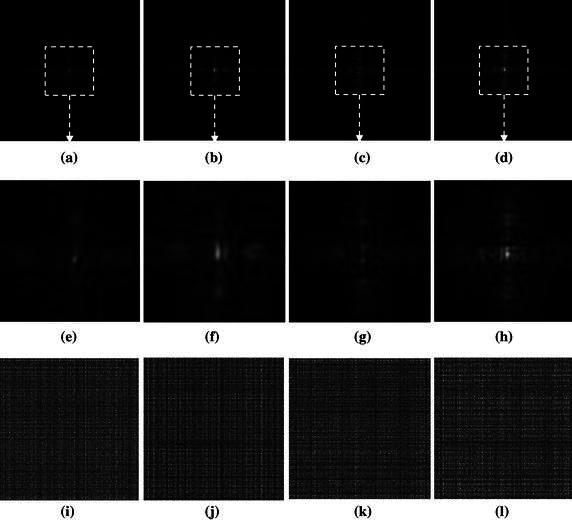
The first row show the magnitude of the Fresnelet coefficients applied to the USAF target image as shown in Fig. 2 with key parameter $d_{1} = 1\;{\text{m}}$. **a** The approximation data, **b** the *horizontal* detail data, **c** the *vertical* detail data, **d** the *diagonal* detail data. The *second row* **e**, **f**, **g**, and **h** represents the zoomed-in views of the marked areas of the corresponding images in the first row. The *last row* **i**, **j**, **k**, and **l** shows the corresponding magnitude of the inverse Fresnelet transformed data from the four subbands of the images in the *first row* with key parameter $d_{2} = 0.01\;{\text{cm}}$

**Fig. 2:**
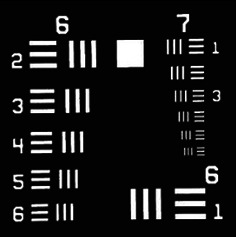
USAF information image for data hiding

Where the low-passed data is represented by the coefficient $f_{\tau ,d}^{{\left( {ll} \right)}}$ and the high-passed detail data are represented by the coefficients $f_{\tau ,d}^{{\left( {lh} \right)}}$, $f_{\tau ,d}^{{\left( {hl} \right)}}$ and $f_{\tau ,d}^{{\left( {hh} \right)}}$, respectively. Figure 1 indicates the Fresnelet coefficients that are acquired from the information data as shown in Fig. 2. It is worth mentioning in Fig. 1 that instead of using the Fresnel transform simple application (Liebling et al. 2003), the information data can be processed by the forward Fresnelet transform so that meaningful information are totally encrypted into dummy image data with four different bands in the form of complex data (Liebling et al. 2003). The magnitudes of the Fresnelet coefficients of the USAF information image are shown in Fig. 1.

**Fig. 3:**
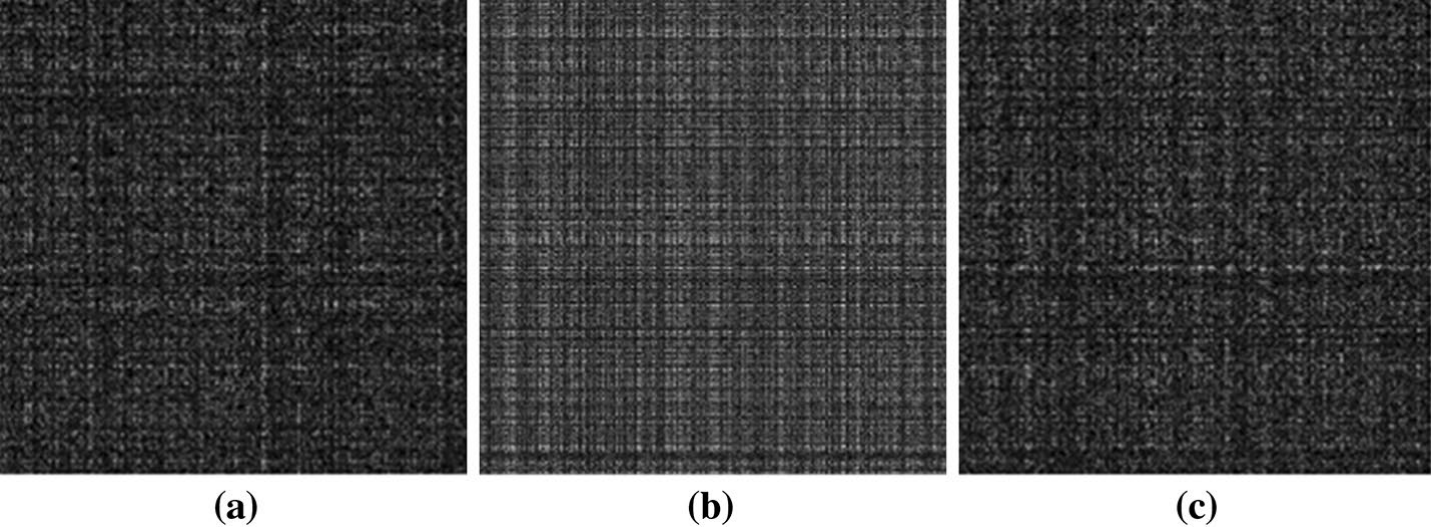
The scrambled data of the information image of Fig. 2 that is reconstructed from the combination of all subband data in the *last row* of Fig. 1 is represented in **a**. It’s intensity is considered as the magnitude of the complex scrambled valued data. The **b** and **c** images are the real and imaginary parts of the scrambled data as shown in **a**

Note that by unitary property of the Fresnelet transform, a reconstruction of an information data can be obtained by applying the conjugate transpose of the forward Fresnelet transform. In this case, the reconstruction has a complex valued data form. The first row of Fig. 1 is the decomposition stage of information data (USAF image shown in Fig. 2) into 4 subbands on employing the application of Fresnelet transform using the distance parameter *d*_1_ and considered as Forward Fresnelet transform propogation. The second row show the central position of information data diffusion with zoomed-in position to show the complete deformation of information data into dummy data (scrambled data). The third row is the inverse propagation of the respective subbands of row one, using the distance parameter *d*_2_, and considered as inverse Fresnelet transform. Figure 3a in the proposed method shows the reconstruction (merging) of four subbands of dummy data from Fig. 1 into single complex data image. Complex property of the Fresnelet transform is the multiresolution property as described in Liebling and Unser (2004). For transmitting, the complex data image (e.g. *a* + *ib*) is separated into two parts; where $a$ and $b$ are real numbers and $i$ (imaginary unit) $i^{2} = \left[ { - 1} \right]$. For transmitting the information data in digital form, we need to separate the complex data as shown in first image of Fig. 3 into real part $a$ (second image of Fig. 3) and magnitude of imaginary part $b$ (last image of Fig. 3). On extraction stage, just by multiplying the magnitude of imaginary part $b$ with $i$, we can reconstruct our required complex data, for getting the extraction image using inverse process.

To communicate the information data with high privacy and improved secrecy, the proposed algorithm uses the Fresnelet transform that takes into account the wavelength and the distance parameters as keys, which are essential for reconstructing the accurate information data. Moreover, the original form of the information data is attained in the reconstruction phase with the exact keys using the inverse Fresnelet transform processes.

## Proposed method

For data handling, we use the wavelet transform (WT) for decomposing and reconstructing the cover image. The wavelet transform is performed with the lifting framework (Mallat 2008) that has low complexity of computation without extra storage cost. For our numerical simulation, the Haar wavelet transform is employed. The proposed data hiding process consists of two steps: embedding process and extraction process.

### The embedding process

The embedding process for data hiding has two starting points. The first is for the encryption of the information image data and the second is for the cover image data in which the encrypted data from the information image is embedded as shown in Fig. 3. To keep the confidential information preservation, the information data is decomposed by employing the Fresnelet transform based on the Haar wavelet. At first, the information image data $f$ is propagated by the Fresnelet transform $F_{\tau }$ with the first distance parameter key, *d*_*1*_ = 1 m, as follow:

9 $$ F_{\tau } \left( {f,d_{1} } \right) = \left( {\begin{array}{*{20}c} {f_{{\tau ,d_{1} }}^{{\left( {ll} \right)}} } &\quad {f_{{\tau ,d_{1} }}^{{\left( {hl} \right)}} } \\ {f_{{\tau ,d_{1} }}^{{\left( {lh} \right)}} } & \quad {f_{{\tau ,d_{1} }}^{{\left( {hh} \right)}} } \\ \end{array} } \right) $$

In the next stage, we generate a scrambled data $D$ from the decomposed data of $f$ by using the inverse Fresnelet transform $IF_{\tau }$ with the second distance parameter key, *d*_2_ = 10^−4^ m, as follow:

10 $$ D = IF_{\tau } \left\{ {\left( {\begin{array}{*{20}c} {f_{{\tau ,d_{1} }}^{{\left( {ll} \right)}} } & \quad  {f_{{\tau ,d_{1} }}^{{\left( {hl} \right)}} } \\ {f_{{\tau ,d_{1} }}^{{\left( {lh} \right)}} } & \quad {f_{{\tau ,d_{1} }}^{{\left( {hh} \right)}} } \\ \end{array} } \right),\, d_{2} } \right\} $$

The scrambled data from the USAF information image are shown in Fig. 1. The scrambled data are complex valued data because of the nature of the Fresnelet transform. We can separate this complex data into the real part $D_{re}$ and the imaginary part $D_{im}$ for embedding those data into suitable detail subbands of the decomposed cover data as (13) and (14). For a given cover image $C$, the wavelet transform (WT) is used for obtaining the subband images in which the coded information data will be embedded. Let $j$ be the finest resolution level of the cover image. By the one level decomposition of $C$, four subband images at the coarser resolution level $j - 1$ are obtained as follows:

11 $$ WT\left( C \right) = \left( {\begin{array}{*{20}c} {C_{j - 1}^{{\left( {ll} \right)}} } & \quad {C_{j - 1}^{{\left( {hl} \right)}} } \\ {C_{j - 1}^{{\left( {lh} \right)}} } & \quad  {C_{j - 1}^{{\left( {hh} \right)}} } \\ \end{array} } \right) $$

By applying the low-pass wavelet filter along the rows and columns of $C$, an approximated data $C_{j - 1}^{{\left( {ll} \right)}}$ is obtained. A horizontally oriented detail image data $C_{j - 1}^{{\left( {lh} \right)}}$ is generated by applying the low-pass wavelet filter along the rows and the high-pass wavelet filter along the columns of $C$. Similarly, a vertically oriented detail image $C_{j - 1}^{{\left( {hl} \right)}}$ is obtained. By applying the high-pass wavelet filter along the rows and columns of $C$, a detailed image data $C_{j - 1}^{{\left( {hh} \right)}}$ is also obtained.

Notice that the approximated data $C_{j - 1}^{{\left( {ll} \right)}}$ is the low-pass subband image data containing high energy. We magnify it to the size of the original cover image by using bi-cubic interpolation and discard all high-passed details $C_{j - 1}^{{\left( {hl} \right)}}$, $C_{j - 1}^{{\left( {lh} \right)}}$, and $C_{j - 1}^{{\left( {hh} \right)}}$. The resized data $R$ of $C_{j - 1}^{{\left( {ll} \right)}}$ is again decomposed into four subbands by using the WT.

12 $$ WT\left( {R  } \right) = \left( {\begin{array}{*{20}c} {R_{j - 1}^{{\left( {ll} \right)}} } & \quad  {R_{j - 1}^{{\left( {hl} \right)}} } \\ {R_{j - 1}^{{\left( {lh} \right)}} } & \quad {R_{j - 1}^{{\left( {hh} \right)}} } \\ \end{array} } \right) $$

The subband data $R_{j - 1}^{{\left( {ll} \right)}}$, $R_{j - 1}^{{\left( {lh} \right)}}$, $R_{j - 1}^{{\left( {hl} \right)}}$, and $R_{j - 1}^{{\left( {hh} \right)}}$ are the low-passed image data, the horizontal detail image data, the vertical detail image data, and the diagonal detail image data, respectively. Note that the significant coefficients in the high-passed subband data are corresponding to edges, corners, and textures. So, we embed the scrambled information image data into the subband data $R_{j - 1}^{{\left( {hl} \right)}}$ and $R_{j - 1}^{{\left( {lh} \right)}}$. The real part $D_{re}$ of the scrambled data is embedded into the subband data $R_{j - 1}^{{\left( {hl} \right)}}$, whereas the imaginary part $D_{im}$ of the scrambled data is embedded into the subband data $R_{j - 1}^{{\left( {lh} \right)}}$ as (13) and (14). A scale parameter $\alpha$ with values between 0 and 1 is introduced as a strength factor that controls the weight of embedding of scrambled information data. In the proposed method, we use an empirical value $\alpha = 0.05$ of the scaling factor for embedding the dummy information data as follow:

13 $$ \tilde{R}_{j - 1}^{{\left( {hl} \right)}} = R_{j - 1}^{{\left( {hl} \right)}} + \alpha D_{re} $$

14 $$ \tilde{R}_{j - 1}^{{\left( {lh} \right)}} = R_{j - 1}^{{\left( {lh} \right)}} + \alpha D_{im} $$

where $\tilde{R}_{j - 1}^{{\left( {hl} \right)}}$ and $\tilde{R}_{j - 1}^{{\left( {lh} \right)}}$ are the modified subband data containing the dummy information data. Instead of using the resized data $R_{j - 1}^{{\left( {ll} \right)}}$ during the reconstruction process, we use the approximate data $C_{j - 1}^{{\left( {ll} \right)}}$ of the original cover image for achieving better imperceptibility and a reliable extraction of the embedded dummy information.

15 $$ E = IWT\left( {\begin{array}{*{20}c} {C_{j - 1}^{{\left( {ll} \right)}} } & {\tilde{R}_{j - 1}^{{\left( {hl} \right)}} } \\ {\tilde{R}_{j - 1}^{{\left( {lh} \right)}} } & {R_{j - 1}^{{\left( {hh} \right)}} } \\ \end{array} } \right) . $$

The above reconstruction process with the inverse wavelet transform (IWT) provides an information embedded image $E$ after embedding the real and imaginary parts of $D$ in the specified bands of $C$.

### The extraction process $E^{{\left( {ll} \right)}}$

**Fig. 4:**
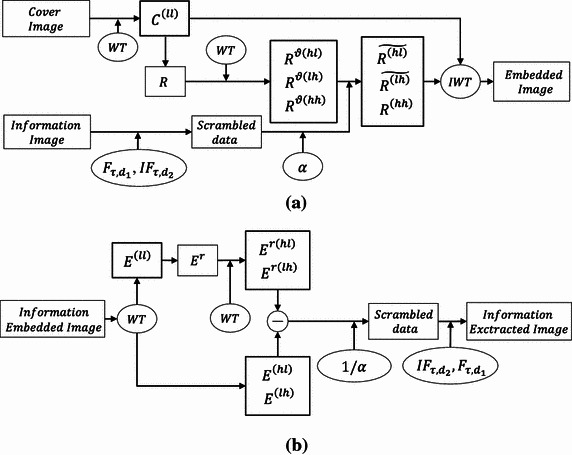
Diagram showing **a** the embedding process and **b** the extraction process

The extraction process is the reverse of the embedding process as shown in Fig. 4. The wavelet transform (WT) is used to decompose the information embedded image $E$ into four subband data sets $E_{j - 1}^{{\left( {ll} \right)}}$, $E_{j - 1}^{{\left( {hl} \right)}}$, $E_{j - 1}^{{\left( {lh} \right)}}$, and $E_{j - 1}^{{\left( {hh} \right)}}$. The high frequency subband data $E_{j - 1}^{{\left( {hl} \right)}}$ and $E_{j - 1}^{{\left( {lh} \right)}}$ are information carrier data sets that are preserved in the same position. By using the bi-cubic interpolation, we resize the low-pass subband data $E_{j - 1}^{{\left( {ll} \right)}}$ with the same size as that of the input embedded image. In order to extract the embedded information data, the WT is applied again to the resized data $E_{{}}^{r}$ and then we obtain the following four subband data $E_{j - 1}^{{r\left( {ll} \right)}}$, $E_{j - 1}^{{r\left( {hl} \right)}}$, $E_{j - 1}^{{r\left( {lh} \right)}}$, and $E_{j - 1}^{{r\left( {hh} \right)}}$.

The scrambled data (with real and imaginary parts) can be extracted by subtracting the high frequency subband data $E_{j - 1}^{{r\left( {hl} \right)}}$ and $E_{j - 1}^{{r\left( {lh} \right)}}$ from the data $E_{j - 1}^{{\left( {hl} \right)}}$ and $E_{j - 1}^{{\left( {lh} \right)}}$ of the information embedded image, respectively. Afterwards the difference net data are divided by $\alpha$ so that the scrambled information data is obtained. The extracted real and imaginary parts of the scrambled data are reunited in the form of complex data. Finally, this complex scrambled data is processed by the inverse Fresnelet transforms using the same parameter keys of the Fresnelet transform in order to get the secret information image.

## Simulation and evaluation

**Fig. 5:**
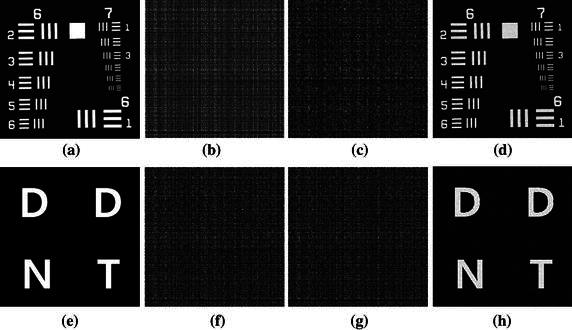
In the *first row*, **a** is the original USAF information image, **b** and **c** images are the real and imaginary parts of the encoded USAF information data by the Fresnelet. The **d** is the extracted information image from the information embedded Lena image. In the *second row*, **e**, **f**, **g**, and **h** show the corresponding result of the *first row*: **a**–**d** with DDNT information image from the information embedded Lena image, respectively

An information image data of size $N \times N \left( {N = 256} \right)$ is embedded into a cover image of size $2N \times 2N$. In the proposed method, we consider a wavelength $\lambda = 632.8\;{\text{nm}}$ (${\text{nm}}\;{ = }\;{\text{nanometer}}$), a sampling interval size ∆ = 10 nm of a hypothetical CCD plane, and distances $d_{1} = 1\;{\text{m}}$ (${\text{m}}\;{ = }\;{\text{meter}}$) and $d_{2} = 10^{ - 4} \;{\text{m}}$ (Nazeer et al. 2013). These parameters are employed in the Fresnelet transform operations for the embedding and extraction phases and are considered as the key parameters. The extracted information images are estimated by the measurement of correlation coefficients (CC) with the original information image data. In Table 1, we provide the simulation result that shows the CC values of the extracted information images from various information embedded images according to the scaling factors. Figure 5 shows the original information images (USAF, DDNT), the encoded information data, and the extracted information.

**Table 1:** CC index of the extracted information image

Information embedded images (512 × 512)	USAF (256 × 256) Scaling factor (*α*)					DDNT (256 × 256) Scaling factor (*α*)				
	0.03	0.04	0.05	0.06	0.07	0.03	0.04	0.05	0.06	0.07
Camerman	0.9873	0.9920	0.9943	0.9956	0.9965	0.9886	0.9935	0.9957	0.9970	0.9977
Pepper	0.9877	0.9924	0.9946	0.9960	0.9968	0.9889	0.9938	0.9959	0.9971	0.9978
Elaine	0.9875	0.9922	0.9946	0.9960	0.9969	0.9888	0.9936	0.9960	0.9972	0.9979
Lena	0.9877	0.9923	0.9947	0.9969	0.9960	0.9888	0.9937	0.9960	0.9972	0.9980

**Fig. 6:**
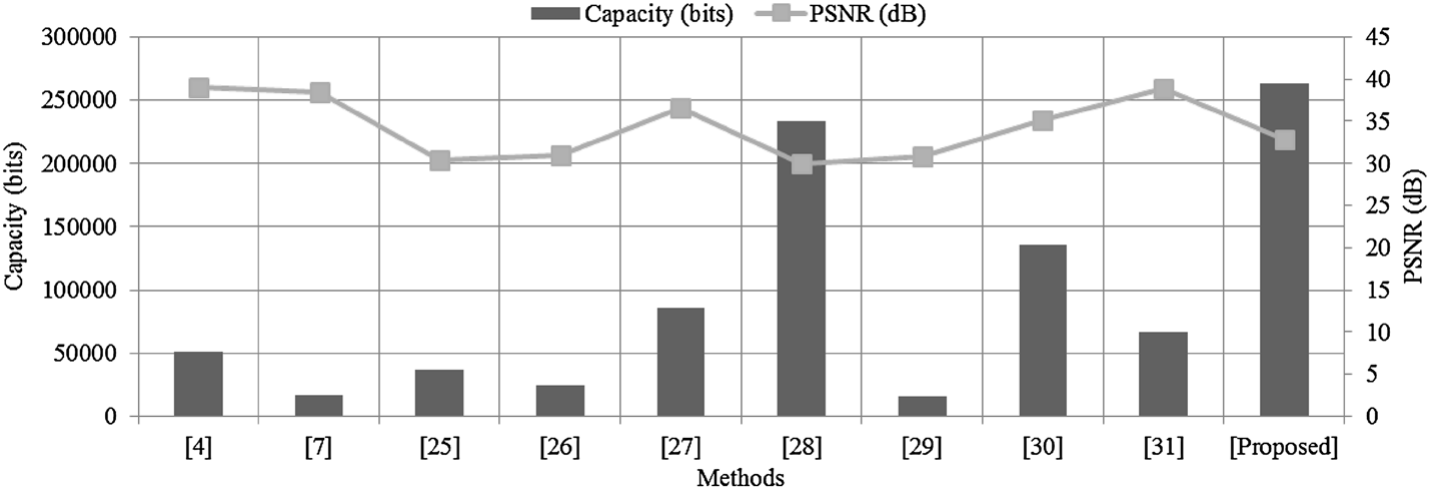
Graphical demonstration of the comparison of Table 2

**Fig. 7:**
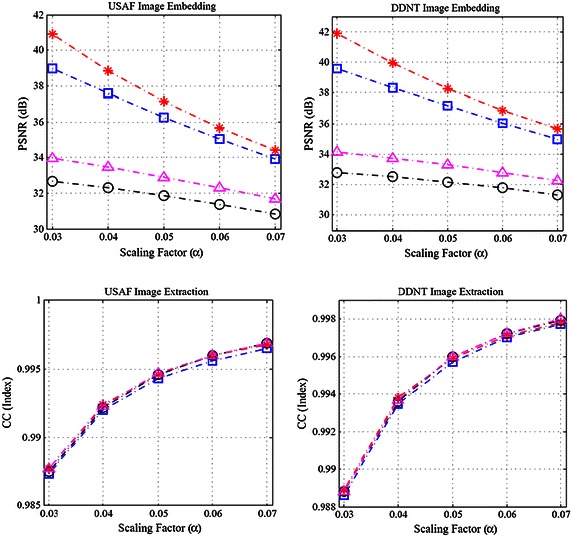
We use the four benchmark images: “*red asterisk*” Cameram; “*open circle*” Peppers; “*pink triangle*” Elaine; “*unfilled blue square with middle dot*” Lena. Simulation results with various scaling factors: $\alpha = 0.03$, $0.04$, $0.05$, $0.06$, $0.07$. For the test cover image data. In the *first row*, the *left plot* shows the quality (PSNR) of the information embedded images corresponding benchmark images after embedding the USAF. Similarly, the *right plot* is for the case of DDNT. In the *second row*, the *left plot* shows the CC values of extracted USAF information data from the information embedded images above. Similarly, the *right plot* is for the case of DDNT

To analyze the presented algorithm, two separate modules of MATLAB are developed for the two processes: embedding and extraction. The numerical evaluation of the performance of our scheme is accomplished by using the CC value and the peak signal-to-noise ratio (PSNR) (Li and Dai 2009). The experiments demonstrate that the proposed method provides better imperceptibility of the information data with large capacity [the payload size in bits (Chen et al. 2010)] as compared with other methods as shown in Table 2 and Fig. 6. Notice from Table 2 and Fig. 6 that the proposed method offers higher embedding capacity of information data than other methods. Note that the two measurements (PSNR, the payload size) are known as the most commonly used criteria for evaluating the performance of reversible data hiding techniques. We use the standard benchmark Lena image as a cover image data for comparison of the performance. Also, various different cover images are used for testing the performance of our proposed method in terms of hiding data capacity and CC as shown in Tables 1, 3 and Fig. 7.

**Table 2:** Comparison of the information embedded image quality and the capacity of embedded information

Cover images	Lena (size: 512 by 512)	
Methods	Capacity (bits)	PSNR (dB)
Wu et al. (2005)	51,219	*38.94*
Ashourian and Ho (2005)	16,384	*38.50*
Chang et al. (2006)	24,360	*30.94*
Chen et al. (2010)	36,850	30.34
Yang (2010)	85,507	*36.60*
Tian (2003)	233,067	29.97
Lin and Zhang (2012)	16,129	*30.78*
Kamstra and Heijmans (2005)	135,547	*35.20*
Luo et al. (2010)	66,064	*38.80*
Proposed method	*263*,*222*	32.90

**Table 3:** PSNR (dB) of the information embedded image

Information embedded images (512 × 512)	USAF (256 × 256) Scaling factor (*α*)					DDNT (256 × 256) Scaling factor (*α*)				
	0.03	0.04	0.05	0.06	0.07	0.03	0.04	0.05	0.06	0.07
Camerman	38.99	37.57	36.24	35.03	33.94	39.60	38.35	37.15	36.03	35.00
Pepper	40.92	38.85	37.14	35.68	34.42	41.89	39.94	38.29	36.88	35.64
Elaine	32.67	32.29	31.85	31.36	30.85	32.80	32.51	32.16	31.77	31.35
Lena	33.97	33.46	32.90	31.66	32.29	34.14	33.75	33.30	32.80	32.27

Table 2 and Fig. 6 shows the comparison of our results with those of existing techniques. Chang et al. (2006) presented a reversible data hiding method based on the SMVQ compression codes. Yang (2010) suggested a method based on the wavelet transform and produced large capacity in high details. Tian (2003) suggested a large capacity reversible data hiding method that is based on the difference-expansion (DE). The aim of Lin’s method (Lin and Zhang 2012) was to gain a data-reading approach. Afterwards, various methods, such as Kamstra method (Kamstra and Heijmans 2005) and Luo’s method (Luo et al. 2010), and Ashourian’s method (Ashourian and Ho 2005) have been reported to raise the capacity of the embedding data while minimize to the distortion. For (Yang 2010; Tian 2003; Lin and Zhang 2012; Kamstra and Heijmans 2005), the main reason of the high quality of the information embedded image in comparison with our proposed method is that all of them are non-blind data hiding techniques, where the original cover image needs to be used to extract the embedded information. So, the embedding process of a non-blind method can be optimally designed with respect to the good quality of the information embedded data. On the other hand, a blind data hiding is defined as the method without using any clue of original cover image data in the extraction process. So, the embedding process of a blind hiding method would be accordingly designed with concerning an efficient extraction process. In this case, the information embedded image data has relatively lower quality as compared with the non-blind method.

Note that there is a trade-off between the capacity of an embedded information data and the quality of the information embedded image (Lin and Zhang 2012). For our proposed method, the capacity of an embedded information data is approximately twice as big as that of the information embedded data. Thanks to the diffusion process of the Fresnelet transform applied to the information data, we can generate a coded data with almost uniformly scattered structure (Nazeer et al. 2013; Zhou et al. 2014) as shown in Figs. 1, 5, which would be embedded into a cover image with keeping good quality of the information embedded data. While, for most of other existing techniques (Wu et al. 2005; Ashourian and Ho 2005; Chen et al. 2010; Chang et al. 2006; Yang 2010; Tian 2003; Chen and Huang 2014; Lin and Zhang 2012; Kamstra and Heijmans 2005; Luo et al. 2010), the information data of small capacity is hidden in a cover image data without any diffusion process as shown in Table 2 and Fig. 6. Since it is not possible to simultaneously maximize the robustness or imperceptiveness of the information embedded data and the capacity of the information data image, therefore an adequate balance of these two features for a specific application (Chen et al. 2010; Chang et al. 2006; Yang 2010; Chen and Huang 2014) is developed. In this regard, a data hiding method would forgo the low imperceptibility and the robustness in favor of large capacity of the embedded image (Nazeer and Kim 2012). On the other hand, an invisible watermarking method, which may not contain large capacity of a watermark, would definitely favor the high imperceptibility and the robustness of the embedded image (Wu et al. 2005; Huang et al. 2011; Shang 2011, 2015).

With the numerical simulation, we empirically analyze the choice of the scaling factor $\alpha$. Since the scaling factor is weighting value for the information data, the different values of $\alpha$ in the range of 0–1 are affecting the quality of the information embedded image and the CC value of the extracted information data. In our simulation, we use the values $\alpha = 0.03$, $0.04$, $0.05$, $0.06$, $0.07$ that give a consistent result of PSNR and CC values as shown in Fig. 7. In this case, we employ four benchmark cover image data: Cameraman, Peppers, Elaine, Lena with size 512 × 512 and two benchmark information image data: USAF, DDNT information data with 256 × 256. Note that as increasing the scaling factor, the PSNRs of the information embedded images are decreasing and the CC values of the extracted information data are increasing and shown in Fig. 7. We focus on the value $\alpha = 0.05$ which provides the optimal result for the trade-off between the imperceptibility of information embedded images and the CC values of the extracted information data for all four graphs in Fig. 7.

## Conclusions

This paper proposes a blind data hiding approach which has improved imperceptibility of the embedded information data as well as high capacity. The proposed method is a relatively new approach for two reasons. First, the Fresnelet transform is employed for scrambling the information image data with different distance parameters as keys, while maintaining the overall energy of the information data in the form of dummy complex data to be embedded in the cover image data. Second, the information data is extracted blindly in the extraction phase without the need of the original cover image data. Furthermore, our proposed method is useful for maintaining reasonable perceptual transparency of an information embedded image with large capacity of information data.
